# Phloem-Triggered Virus-Induced Gene Silencing Using a Recombinant Polerovirus

**DOI:** 10.3389/fmicb.2018.02449

**Published:** 2018-10-23

**Authors:** Diane Bortolamiol-Bécet, Baptiste Monsion, Sophie Chapuis, Kamal Hleibieh, Danièle Scheidecker, Abdelmalek Alioua, Florent Bogaert, Frédéric Revers, Véronique Brault, Véronique Ziegler-Graff

**Affiliations:** ^1^Institut de biologie moléculaire des plantes, CNRS-UPR 2357, Université de Strasbourg, Strasbourg, France; ^2^Architecture et Réactivité de l’ARN, Institut de biologie moléculaire et cellulaire CNRS-UPR 9002, Université de Strasbourg, Strasbourg, France; ^3^UMR1161 Virologie, INRA, ANSES, Ecole Nationale Vétérinaire d’Alfort, Maisons-Alfort, France; ^4^SVQV, INRA UMR 1131, Université de Strasbourg, Colmar, France; ^5^BFP, INRA UMR 1332, Univ. Bordeaux, Villenave d’Ornon, France; ^6^BIOGECO, INRA UMR 1202, Univ. Bordeaux, Pessac, France

**Keywords:** polerovirus, VIGS, phloem, suppressor of RNA silencing, visual monitoring of infection

## Abstract

The phloem-limited poleroviruses infect *Arabidopsis thaliana* without causing noticeable disease symptoms. In order to facilitate visual infection identification, we developed virus-induced gene silencing (VIGS) vectors derived from Turnip yellows virus (TuYV). Short sequences from the host gene *AtCHLI1* required for chlorophyll biosynthesis [42 nucleotides in sense or antisense orientation or as an inverted-repeat (IR), or an 81 nucleotide sense fragment] were inserted into the 3′ non-coding region of the TuYV genome to screen for the most efficient and robust silencing vector. All recombinant viruses produced a clear vein chlorosis phenotype on infected *Arabidopsis* plants due to the expression inhibition of the *AtCHLI1* gene. The introduction of a sense-oriented sequence into TuYV genome resulted in a virus exhibiting a more sustainable chlorosis than the virus containing an IR of the same length. This observation was correlated with a higher stability of the sense sequence insertion in the viral genome. In order to evaluate the impact of the TuYV silencing suppressor P0 in the VIGS mechanism a P0 knock-out mutation was introduced into the recombinant TuYV viruses. They induced a similar but milder vein clearing phenotype due to lower viral accumulation. This indicates that P0 does not hinder the performances of the TuYV silencing effect and confirms that in the viral infection context, P0 has no major impact on the production, propagation and action of the short distance silencing signal in phloem cells. Finally, we showed that TuYV can be used to strongly silence the phloem specific *AtRTM1* gene. The TuYV-derived VIGS vectors therefore represent powerful tools to easily detect and monitor TuYV in infected plants and conduct functional analysis of phloem-restricted genes. Moreover this example indicates the potential of poleroviruses for use in functional genomic studies of agronomic plants.

## Introduction

Virus-induced gene silencing (VIGS) is a valuable tool to produce rapid gene knockdown phenotypes that can be used in genetic approaches to assess plant gene functions (Baulcombe, 1999; Ratcliff et al., 2001; Waterhouse and Helliwell, 2003; Burch-Smith et al., 2004; Senthil-Kumar and Mysore, 2011; Ramegowda et al., 2014). VIGS exploits RNA silencing machinery, a natural antiviral defense that is induced by the perception of double stranded RNA (dsRNA) and provokes targeted RNA degradation, resulting in silencing of the host gene. Small RNA (sRNA) are generated from dsRNA precursors, that can be viral replicating genomes, by the action of Dicer proteins and loaded into an effector complex containing ARGONAUTE 1 (AGO1) which then guides sequence-specific degradation of complementary transcripts, i.e., the cellular target mRNAs (Ding and Voinnet, 2007). VIGS can potentially target any gene after inserting part of the gene sequence into a viral vector. This approach has been successfully applied to study gene loss-of-function in many dicotyledonous (Waterhouse and Helliwell, 2003; Burch-Smith et al., 2004; Golenberg et al., 2009), monocotyledonous plants (Scofield and Nelson, 2009; Mei et al., 2016; Ding et al., 2017) or both (Liou et al., 2014), using RNA or DNA viruses (Burch-Smith et al., 2004; Kumar et al., 2014; Kant and Dasgupta, 2017).

In this work we used VIGS as a tool to enable visualization of polerovirus infection in *Arabidopsis thaliana* (*A. thaliana*). Indeed most poleroviruses infect *A. thaliana* without inducing any symptoms. Discrimination between infected and non-infected plants requires subsequent expensive and time consuming molecular or serological analyses incompatible with high scale screenings.

Poleroviruses like the other members of the *Luteoviridae* family are restricted to the plant vasculature (Mayo and Ziegler-Graff, 1996). They are confined to phloem cells (companion cells, sieve elements, and phloem parenchyma cells). In nature, poleroviruses are delivered into phloem cells by aphid vectors while in the laboratory they can be inoculated through agroinfection by *Agrobacterium tumefaciens* harboring a binary vector containing the viral cDNA (Leiser et al., 1992; Prüfer et al., 1995; Lee et al., 2005; Zhang et al., 2009; Delfosse et al., 2013; Klein et al., 2014). The polerovirus genome consists of a monopartite positive-sense single-stranded RNA that is packaged into isometric particles. It contains seven to eight ORFs, some of them overlapping extensively and most being essential for systemic infection and aphid transmission (Brault et al., 1995; Chay et al., 1996; Ziegler-Graff et al., 1996; Bruyère et al., 1997; Ashoub et al., 1998; Smirnova et al., 2015). Therefore, introduction of large sequences into polerovirus genomes has remained a challenge for many years as recombinant infectious clones were unable to invade non-inoculated leaves or showed deletions in the inserted sequence (Nurkiyanova et al., 2000). This is likely due to genome size constraints to package appropriately the viral genome into icosahedral particles. Only very recently Boissinot et al. (2017) obtained a recombinant polerovirus derived from Turnip yellows virus (TuYV) expressing EGFP in fusion with the non-structural domain of the readthrough protein. TuYV-EGFP virus was visualized by fluorescence in the vasculature of infected plants. However, following TuYV-EGFP infection *in planta* requires UV-emitting equipment which is not compatible with easy recording and high throughput screening.

In this report, we engineered and analyzed several modified TuYV genomes containing small size inserts able to silence the host *CHLI1* gene involved in chlorophyll synthesis, thus allowing fast and reliable monitoring of infection by the development of vascular-specific yellowing symptoms. We compared the silencing efficiency and durability induced by the different TuYV constructs by visual monitoring of symptoms and by quantitative RT-PCR analysis. We identified a construct that conserved both insert and genome integrity after aphid transmission. As TuYV encodes the strong RNA silencing suppressor (RSS) P0 (Pazhouhandeh et al., 2006; Bortolamiol et al., 2007) we assessed its potential role in modulating silencing efficiency of the targeted gene. Finally we further proved that TuYV phloem restriction can be used to efficiently silence a phloem specific host gene, *RTM1*, a gene involved in resistance to long distance movement of potyviruses in *A. thaliana* Col-0 (Chisholm et al., 2001; Revers and Garcia, 2015). This study thus demonstrates that the polerovirus TuYV can be engineered into a powerful silencing vector able to specifically target genes expressed in phloem cells.

## Materials and Methods

### Construction of TuYV Recombinants

**FIGURE 1:**
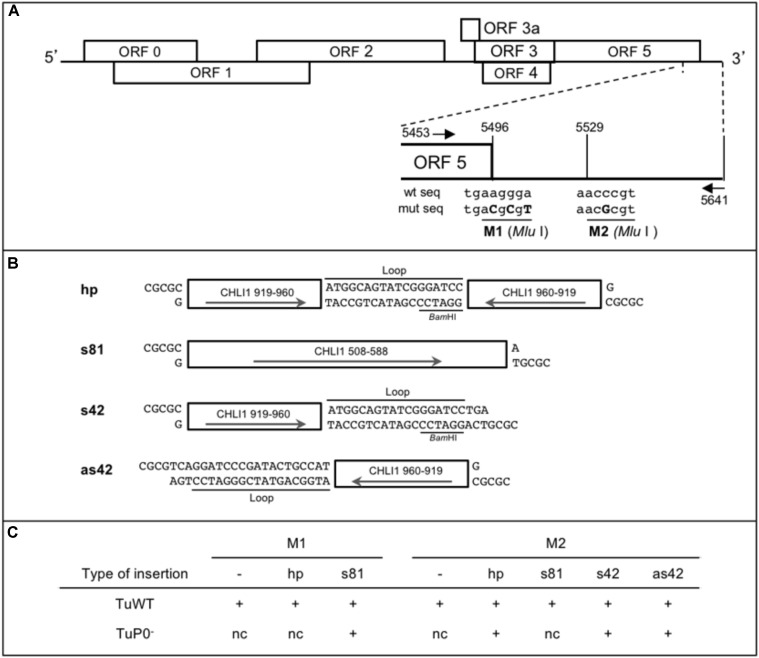
Design of CHLI1 constructs and their sequences introduced in TuYV recombinant viruses used as VIGS vectors. **(A)** Schematic representation of the TuYV genome and position of the two *Mlu*I restriction sites (M1 and M2) in the 3′ end of the TuYV genome. The mutated nucleotides introduced in the virus genome are shown in capital letters. The arrows locate the position of the primers (Tu-5453-Fw and Tu-5641-Rev) used to perform the PCR reactions to analyze the viral progeny in infected plants (Supplementary Table S1). **(B)** Description of the *CHLI1* fragments inserted into one of the *Mlu*I sites to produce the recombinant viruses. hp, hairpin sequence; s81, sense sequence of 81 nucleotides (nts); s42, sense sequence of 42 nts; as42, antisense sequence of the same 42 nts. s42 and as42 contain in addition a 18 nts heterologous sequence annotated “Loop” that contributed to the construction of the hp clone. Arrows indicate the orientation of the inserts. All DNA fragments have *Mlu*I compatible cohesive ends. Position of the *Bam*HI site in the loop sequence is indicated. **(C)** Overview of the viral constructs. “+” indicates the recombinant viruses that were generated. M1 and M2 refer to the restriction sites used to introduce the different *CHLI1* fragments. “–” refers to the viral mutants containing only the above indicated *Mlu*I restriction site with no additional sequence. TuWT and TuP0^-^ are the viral backbones in which the different fragments were inserted. nc, clone not constructed.

Turnip yellows virus recombinants are derived from the full-length cDNA clone of the wild-type TuYV [pBinTuWT, formerly pBinBW_0_ (Leiser et al., 1992)]. They have been obtained by subcloning the 3′ part of the viral genome in pKS vector [Figure 1A; primers Tu-5351-Fw and Bin-Tu-Rev, Supplementary Table S1; (Bortolamiol, 2008)]. The previously described M1 mutant which contains a *Mlu*I site at the end of ORF5 (Brault et al., 1995) served as template for M1-derived construct (pKS.M1) whereas M2 mutation (insertion of a *Mlu*I site in the 3′ non translated sequence of TuYV) was obtained by mutagenic PCR using Mlu2-Dir and Mlu2-Rev primers (Supplementary Table S1) and the pBinBW_0_ vector as template (pKS.M2).

The 81base pairs (bp) sense fragment of *CHLI1* (AT4G18480; cDNA: nts 508-588) was obtained by PCR using the primers CHLI1-508-Fw and CHLI1-588-Rev (Supplementary Table S1) on total cDNA from *A. thaliana* Col-0 and introduced into one of the *Mlu*I sites (M1 or M2) after cutting the PCR fragment with *Bss*HII/*Mlu*I (these two restriction enzymes release compatible overhangs). The 42 bp long fragment (cDNA: nts 919-960) was created by hybridizing the complementary oligonucleotides CHLI1-42-1 and CHLI1-42-2 (Supplementary Table S1) and introduced either in sense (s42) or antisense (as42) orientation into the M2 *Mlu*I site. The hairpin construct was obtained by introducing the hybridized oligonucleotides CHLI1-hp1 and CHLI1-hp2 (Supplementary Table S1) into the s42 clone cut by *Bam*HI and *Mlu*I.

Eight different pKS subclones were generated and the inserts containing the exogenous sequences were introduced by replacement of the corresponding *Hind*III fragment into pbinTuWT or the TuP0^-^ mutant [formerly BW 1.6346 (Ziegler-Graff et al., 1996)]. The first *Hind*III site is located at nucleotide 5366 in the viral cDNA, and the second site is localized 25 nucleotides downstream of the viral sequence in the plasmid sequence (see Figures 1B,C for the list of clones).

To obtain Tu-*RTM1*, an 81 bp fragment from *RTM1* gene (AT1G05760) was amplified by PCR on total cDNA from *A. thaliana* Col-0 using primers RTM1-276-Fw and RTM1-357-Rev (Supplementary Table S1, cDNA: nts 276-357), digested with *Mlu*I and introduced into the *Mlu*I site of pKS.M2. The binary recombinant clone was constructed as described above.

### Plant Material, Agroinoculation, and Aphid Transmission

Wild-type TuYV (TuWT), TuP0^-^ or TuYV recombinant viruses were agroinoculated to 4–5 week-old *A. thaliana* Col-0 plants. *Agrobacterium tumefaciens* harboring binary plasmids were grown to an OD_600nm_ of 0.5 and agroinfiltrated into leaves as described previously except a needleless syringe was used (Leiser et al., 1992). Mock-treated plants were infiltrated with *A. tumefaciens* carrying a pBin19 vector. *Suc*:*SUL-LUS* plants were described by (Himber et al., 2003).

Aphid transmission experiments of TuYV were performed using virus-free *Myzus persicae*. Aphids were fed on agroinoculated plants for 24 h before being transferred to healthy *A. thaliana* (10 aphids/plant). Infection was monitored by DAS-ELISA 18 days after transmission (Bruyère et al., 1997).

### Progeny Analysis by RT-PCR

Total RNA was extracted from randomly collected systemically infected leaves above the infiltrated leaves of *A. thaliana* plants using Tri Reagent^®^ (Sigma) and treated with RNAse-free DNAse (QIAGEN). Reverse transcription of TuYV RNA was performed using a specific oligonucleotide hybridizing to the 3′end of the viral genome Tu-5641-Rev (Supplementary Table S1) and SuperScript^®^ III Reverse Transcriptase (Invitrogen) according to manufacturer’s instructions. The cDNA was amplified by PCR using the above antisense primer and primer Tu-5351-Fw to generate a fragment covering the 3′ end of TuYV RNA containing the insert. Fragments were separated on a 6% acrylamide-bisacrylamide (19/1) gel in TBE 0.5x buffer. Each band was cut out of the gel, individually ground and incubated over night at 37°C in a “crush and soak” buffer (500 mM AcNH_4_, 0.1% SDS, and 0.1 mM EDTA). The filtered DNA recovered in the aqueous phase was precipitated by isopropanol and further purified by phenol-chloroform and precipitated by ethanol before being sequenced.

### Endogenous mRNA and Viral RNA Analysis by Real-Time RT-PCR

Expression of *CHLI1* (AT4G18480) and *RTM1* (AT1G05760) and accumulation of TuYV RNA were analyzed by real-time RT-PCR. To measure *CHLI1* expression, total RNA was extracted 19 dpi from 100 mg of frozen ground tissue from pools of two plants (two to five pools were analyzed/virus) following the TRI Reagent^®^ Protocol with one additional phenol and two chloroform steps before ethanol/sodium acetate precipitation. cDNA was synthesized using a mix of oligo(dT)_18_, Tu-4942-Rev and CHLI1-340Rev primers (listed in Supplementary Table S1), 2 μg of total RNA and SuperScript^®^ III Reverse Transcriptase (Invitrogen). EF1α (AT5G60390) and SAND family protein (AT2G28390) were used as internal reference genes. Real-time RT-PCR was performed using SYBR Green Master mix I (Roche) and specific primers, CHLI-245-Fw and CHLI1-340-Rev and Tu-3694-Fw and Tu-3830-Rev, respectively, for *CHLI1* mRNA and TuYV detection (Supplementary Table S1), on a Lightcycler LC480 apparatus (Roche) according to manufacturer’s instructions. Relative gene expression levels of *CHLI1* and TuYV were calculated by means of the linear regression of efficiency method using LinRegPCR software (version 7.4) and averaged over three technical replicates. After normalization with the reference genes, values were further normalized with two pools of mock-inoculated samples for *CHLI1* expression and three pools of TuWT-infected plants for viral RNA accumulation.

For Tu-*RTM1* VIGS experiments, non-inoculated leaves from TuYV-inoculated plants were randomly collected at 36 dpi. Total RNA was extracted as indicated above from individual plants and treated as before. Primers RTM1-189-Fw and RTM1-319-Rev were used for *RTM1* mRNA detection and Tu-3694-Fw and Tu-3830-Rev for TuYV detection (Supplementary Table S1). Real-time RT-PCR was performed on RNA from individual treated plants (5 or 6) and quantification was performed as described above and data normalized using, as references, Tu-WT for virus quantitation or mock for RTM1 transcript quantitation.

### Co-immunoprecipitation

Purification of the FLAG-AGO1 proteins was performed as described previously (Bortolamiol et al., 2007). The sRNA incorporated into Flag-AGO1 proteins were isolated by Tri Reagent^®^ (Sigma) extraction after immunoprecipitation and run on a 16% polyacrylamide gel containing urea. After transfer to Hybond-NX membrane (GE-Healthcare), RNA were hybridized with a 3′TuYV-specific PCR probe using primers Tu-5280-Fw and Tu-5641-Rev (Supplementary Table S1) ^32^P-radiolabelled by random priming. Proteins (plant total proteins or immunoprecipitated proteins) were denatured in Laemmli buffer × 2 for 8 min at 100°C prior 10% SDS-PAGE (Laemmli, 1970). After transfer to PVDF Immobilon-P membrane (Millipore) AGO1 was detected using a monoclonal anti-FLAG^®^ M2 antibody (Sigma) (Figure 1) or a specific AGO1 antibody (gift from D.C. Baulcombe, Supplementary Figure S1) and TuYV RT protein was detected using a specific polyclonal antibody (Reutenauer et al., 1993).

### Statistical Analysis

To statistically compare the values of multiplication rate of wild-type TuYV with the TuYV recombinants and of *CHLI1* and *RTM1* gene expression in the various plant samples, one-way ANOVA following by Tukey’s *post-hoc* test was applied. All tests were performed using a significance level of α = 0.05. All analyses were produced using the statistical package R software.3.4.1 (R Core Team, 2017).

## Results

### TuYV Is a Target of RNA Silencing and Can Be Used as a Vector in VIGS Experiments

The use of a viral genome as an efficient VIGS vector requires several conditions: (1) the virus has to induce the production of viral small interfering RNAs (vsiRNA) generated by Dicer-like enzymes from both the viral and the host-inserted sequences, (2) the vsiRNA must efficiently target the host mRNA, and (3) the silencing suppressor activity of the engineered virus should not compromise the silencing activity.

A previous study reported that poleroviruses display at least some resistance to the degradation by RNA silencing (Brault et al., 2002). TuYV infection of transgenic *Nicotiana benthamiana* plants expressing a homologous transgene derived from the same virus, triggered efficient RNA silencing of the transgene mRNA while the viral genome was only poorly affected by the plant defense mechanism. We therefore addressed whether TuYV could induce the production of functional vsiRNA acting *in trans* during infection of *A. thaliana*, a prerequisite for its use in VIGS experiments.

**FIGURE 2:**
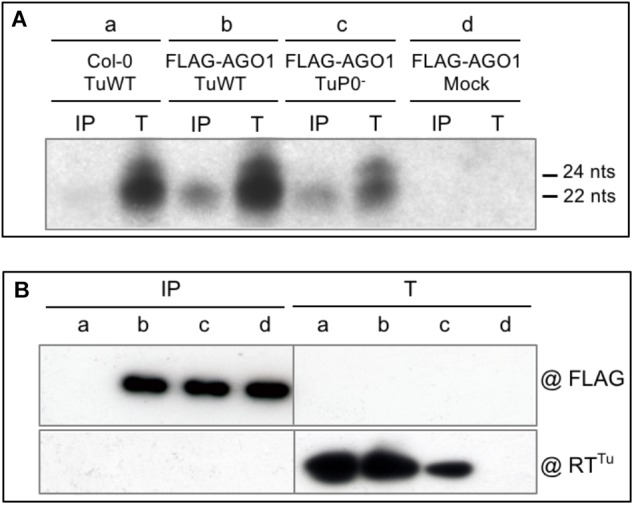
Analysis of vsiRNA generated during TuYV infection. **(A)** Characterization of TuYV-vsiRNA loaded on FLAG-AGO1 protein. Wild-type TuYV (TuWT) (**a** and **b**) or P0-deficient TuYV (TuP0^-^) **(c)** were agroinoculated to *A. thaliana* Col-0 plants **(a)** or *FLAG-AGO1/ago1-36* (FLAG-AGO1) plants (**b**, **c**, and **d**). Plant material was harvested 26 dpi from upper non-inoculated leaves. Total RNA extracted from infected plants (T) or vsiRNA bound to FLAG-AGO1 extracted after immunoprecipitation with FLAG antibodies (IP) were analyzed by northern blot. Hybridization was performed using a TuYV probe corresponding to the 3′ end of genomic RNA. The size of the small RNA is indicated on the right. **(B)** Protein input (T) and immunoprecipitated FLAG-AGO1 proteins using FLAG antibodies (IP) were analyzed by western blot using anti-FLAG antibodies (@FLAG, upper part) or antibodies specific to the TuYV minor capsid protein RT (@RT^Tu^) (lower part).

To analyze the vsiRNA generated during viral infection and potentially loaded on AGO1 we agroinoculated *A. thaliana* plants expressing or not a functional epitope-tagged version of AGO1 [*FLAG-AGO1;* (Baumberger and Baulcombe, 2005)] with wild-type TuYV (TuWT). Three weeks post-infection anti-FLAG immunoprecipitations were performed on systemically infected leaves and both loaded vsiRNA and proteins were analyzed. Total RNA extracts from systemically infected leaves of TuYV-infected Col-0 and *FLAG-AGO1* plants showed a major band of vsiRNA that was retrieved in immunoprecipitated samples from FLAG-AGO1 infected plants and not from control infected Col-0 plants (Figure 2A, compare lanes a-IP and b-IP). This indicates that the shortest and most abundant species of TuYV-derived vsiRNA is efficiently loaded onto AGO1.

Detection of vsiRNA associated to AGO1 was rather unexpected regarding the mode of action of the RNA silencing suppressor P0 encoded by TuYV. Indeed, P0 was shown to act downstream of vsiRNA production by inhibiting the siRNA-guided cleavage activity of the RISC complex (RNA Induced Silencing Complex) (Bortolamiol et al., 2007). In particular, P0 induced the degradation of AGO1, the major actor of the RISC effector, in both transgenic P0 plants and in transient expression experiments (Baumberger and Baulcombe, 2005; Bortolamiol et al., 2007). P0 prevented the *de novo* association of AGO1 with siRNA (Csorba et al., 2010) and led to AGO1 decay through the autophagy pathway (Derrien et al., 2012). To investigate the loading of vsiRNA on AGO1 in the absence of P0, a silencing suppressor P0-deficient TuYV [TuP0^-^, previously referred to as BW1.6346 (Ziegler-Graff et al., 1996)] was agroinoculated on Col-0 and *FLAG-AGO1* plants and TuYV-derived siRNA were analyzed as described above. TuP0^-^-infection of *FLAG-AGO1* plants generated vsiRNA similar to those produced by TuWT that could be loaded onto FLAG-AGO1 (Figure 2A, lane c). This indicates that P0 protein does not prevent siRNA generation nor their loading on AGO1 in the viral infection context. The lower level of vsiRNA observed in these plants is consistent with the reduced accumulation of the P0-deficient virus, as observed in Figure 2B (lane Tc) which shows lower accumulation of the RT protein (minor structural protein) in the TuP0^-^-infected plant extract. Decrease in virus accumulation of a P0 deficient TuYV mutant was previously reported by Ziegler-Graff et al. (1996). In contrast, the level of immunoprecipitated AGO1 is similar in both TuWT- and TuP0^-^-infected and control plants (Figure 2B, lanes IP-b, -c, and -d). However, as P0 is known to induce AGO1 degradation, one could expect a reduced level of FLAG-AGO1 protein in the *FLAG-AGO1* plants infected by TuWT compared to the mock- or TuP0^-^-infected plants. It is conceivable that the IP setting has led to saturation of the antibodies with FLAG-AGO1 proteins, which would mask any subtle difference in FLAG-AGO1 stability between TuWT and TuP0^-^-infected plants. Unfortunately, FLAG-AGO1 proteins are not detected in total protein extracts in the conditions used for this experiment and the expected decrease in the FLAG-AGO1 accumulation in TuWT-infected plants cannot be verified. In other similar experiments (example in Supplementary Figure S1) no clear difference in FLAG-AGO1 accumulation was observed between several TuWT-infected and mock-inoculated Col-0 plants. Such discrepancy with former reports can be explained by the phloem restriction of TuYV and the limited number of infected cells compared to the total of plant cells. A destabilization effect on FLAG-AGO1 solely in phloem cells could therefore be masked by the bulk of proteins that was not affected in the other non-infected cell types.

These experiments demonstrate that virus-derived siRNA are produced in TuWT-infected plants despite the presence of the silencing suppressor P0 and are further incorporated into some AGO1. Such vsiRNA can potentially target the viral RNA and any endogenous mRNA sharing homologous sequences. These results prompted us to design recombinant TuYV viruses to evaluate their silencing activity in VIGS experiments in *A. thaliana*.

### Construction of TuYV-Based VIGS Vectors

Since stable virions are required for TuYV systemic spread in *Nicotiana* species (Brault et al., 2003) and in *A. thaliana* plants (Hipper et al., 2014), the genome size constraint imposed for the formation of stable isometric particles was taken into consideration in the design of the TuYV-based VIGS vectors. Previous studies have shown that inserting long foreign sequences in the genome of poleroviruses could be detrimental to genome stability, systemic movement or aphid transmission of the resulting viruses (Nurkiyanova et al., 2000; Boissinot et al., 2017). In particular the genome integrity of TuYV was seriously affected in *A. thaliana* (Boissinot et al., 2017). Furthermore most of the viral proteins encoded by TuYV are required to establish an efficient systemic proliferation and promote aphid transmission (Brault et al., 1995; Ziegler-Graff et al., 1996; Bruyère et al., 1997; Rodriguez-Medina et al., 2015; Smirnova et al., 2015). To accommodate both the size constraints and the gene essential functions, we inserted small foreign sequences (limited to a maximum of 108 nts) in the 3′ untranslated region (3′UTR) of the TuYV genome. However, since the 3′UTR potentially contains sequences required for replication (Brault et al., 1995) foreign sequences were inserted at two different positions to avoid a putative site-specific deleterious effect on viral multiplication.

**FIGURE 3:**
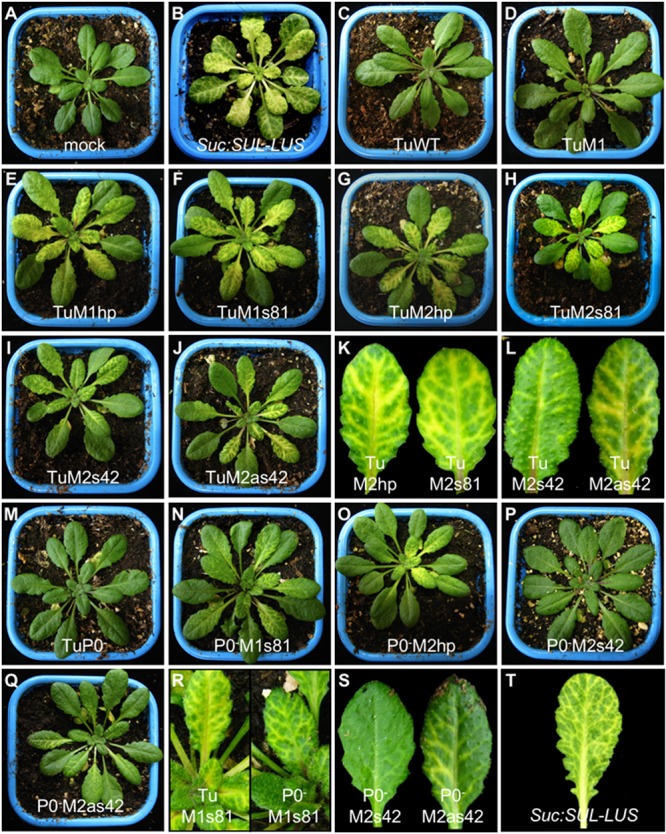
Phenotypic analysis of *A. thaliana* Col-0 plants agroinfected with mutant or recombinant TuYV in either wild-type (panels **C–L**) or P0-deficient mutant virus (P0^-^) background (panels **M–S**). The recombinant viruses are noted in each panel. Pictures were taken 18 days post-inoculation. **(A)** mock-inoculated plant, **(B)** transgenic non-infected *Suc:SUL-LUS* plant (Himber et al., 2003). Plants infected with TuM2 (not shown) were asymptomatic as were plants infected with TuWT and TuM1 (**C** and **D**). Closer views of individual leaves are shown for plants infected with some mutants (**R** and **S**) and the transgenic *Suc:SUL-LUS* plant **(T)**.

Among the most popular endogenous genes that were successfully silenced in VIGS experiments is the *Chlorata 42/CHLI1* gene (Kjemtrup et al., 1998; Turnage et al., 2002) that encodes the nucleotide binding subunit of magnesium chelatase involved in chlorophyll synthesis. Silencing of *CHLI1* gene phenocopies the *sulfur* phenotype of the *Chlorata 42* mutant in *N. benthamiana*, resulting in tissue yellowing. Interestingly, a transgenic line of *A. thaliana* expressing an inverted repeat construct targeting the *CHLI1* gene and placed under the control of the companion cell-specific promoter *AtSUC2* [*Suc:SUL-LUS* (Himber et al., 2003)] exhibits a stable typical yellow chlorosis occurring specifically along the plant vasculature (Figures 3B,T). This indicates that *CHLI1* is expressed in the phloem cells where it can be silenced to give a clear and visible phenotype. Small cDNA fragments of *CHLI1* were therefore inserted into the TuYV genome.

Three different types of inserts deriving from *CHLI1* cDNA were tested and introduced at either M1 or M2 restriction sites (Figure 1A): (i) an inverted repeat (IR) of 42 nts separated by an 18 nts loop sequence that has the potential to fold into a hairpin-like structure (hp) during viral replication was introduced into M1 or M2 sites to generate TuM1hp and TuM2hp, respectively, (Figures 1B,C); (ii) sense and antisense *CHLI1* sequences of 42 nts corresponding to half of the previous IR sequence linked to the loop sequence were introduced at the second *Mlu*I site producing the sense TuM2s42 and antisense TuM2as42 mutants (Figures 1B,C); (iii) a longer insert of 81 nts in sense orientation was introduced into M1 or M2 sites, resulting in the TuM1s81 and TuM2s81 constructs. All of the six aforesaid recombinant viruses were obtained in the wild-type TuYV background. Moreover in order to gain more insight into the role of the RSS P0 in the silencing mechanism targeting the host gene, some of the *CHLI1*-derived inserts were introduced into the P0-deficient TuYV leading to four additional viruses: TuP0^-^M1s81, TuP0^-^M2hp, TuP0^-^M2s42, and TuP0^-^M2as42 (Figure 1C).

### Infectivity and Phenotype Analyses of the Different TuYV Constructs

*Arabidopsis thaliana* Col-0 plants were agroinoculated with the modified TuM1 or TuM2 (containing only the *Mlu*I restriction site) or the various TuM1- and TuM2-derived VIGS constructs. All recombinant viruses were highly infectious with an infection rate ranging between 62.5 and 100% (tested by ELISA for the asymptomatic non-recombinant viruses, data not shown). Whereas TuWT-, TuM1-, and TuM2-infected plants remained symptomless like mock-treated plants (Figures 3A,C,D, data not shown for TuM2), all the recombinant viruses containing a partial sequence of the *CHLI1* gene in the TuWT background developed a vein chlorosis phenotype (Figures 3E–L). This phenotype resembled that exhibited by *Suc:SUL-LUS* plants (Figures 3B,T, Himber et al., 2003) except that the vein chlorosis induced by the TuYV constructs was reproducibly milder compared to *Suc:SUL-LUS* plants. Vein clearing appeared as soon as 9 days post-inoculation (dpi) on young emerging leaves of plants inoculated with the *CHLI1*-recombinant mutants (data not shown). Among all recombinant viruses, those containing the hairpin sequence (hp) or the largest sense construct (s81), whatever the insertion site, produced stronger vein yellowing phenotype on infected plants (Figures 3E–H,K). In contrast, plants challenged with TuM2s42 developed the mildest phenotype (Figures 3I,L). Taken together these observations suggest that TuYV-derived VIGS vectors were all able to trigger silencing of *CHLI1* in phloem cells resulting in vein chlorosis of various intensities.

We also investigated the potential of the four recombinant constructs in the TuP0^-^ background to trigger VIGS, expecting a potential enhancement of the symptoms due to the absence of the RSS P0. All TuP0^-^-derived recombinants were infectious (50–100% of the inoculated plants became infected, tested by ELISA, data not shown) and, as expected and mentioned before, their coat protein antigen titers were significantly lower compared to plants infected with the WT-derived recombinant viruses (Ziegler-Graff et al., 1996). Plants infected with TuP0^-^ remained symptomless (Figure 3M). A vein clearing phenotype appeared with a delay of 3–4 days when compared with plants inoculated with the TuWT-based viruses and the intensity of the vein chlorosis remained milder than that observed in plants infected with the TuWT-based viruses (Figures 3N–S). This difference is likely due to the reduced accumulation of the TuP0^-^ mutants in the infected plants. The observed phenotype was particularly mild in plants infected with the TuP0^-^M2s42 mutant that hardly exhibited any visible phenotype (Figures 3P,S). In summary, the VIGS experiments performed with the silencing suppressor defective mutants showed no intensification of the silencing effect on the endogenous *CHLI1* in the absence of P0.

We then analyzed the speed and the durability of the vein yellowing phenotype induced by the TuYV-recombinant viruses by performing a time course experiment on plants infected with two of the TuYV vectors exhibiting the strongest phenotype, TuM1hp and TuM1s81. The silencing phenotype on TuM1hp-infected plants was visible after a week and slightly faster (1 or 2 days earlier, data not shown) compared to TuM1s81-infected plants and appeared on new developing leaves until 18 dpi (Supplementary Figure S2). For both viruses, source leaves or leaves that were at the source/sink transition state at the time of virus inoculation remained symptomless (Supplementary Figure S2, see red arrows). Three weeks after inoculation, vein chlorosis appeared more scarcely on the new leaves of plants infected with TuM1hp (Supplementary Figure S2A, compare 18, 21, and 28 dpi, white and orange arrows) while the symptoms still developed clearly on new leaves of TuM1s81-infected plants up to 28 dpi (Supplementary Figure S2B, orange arrow). In conclusion, plants infected with TuM1s81 exhibited a more stable and long-lasting silencing phenotype targeting the endogenous *CHLI1* mRNA compared to TuM1hp-infected plants.

**FIGURE 4:**
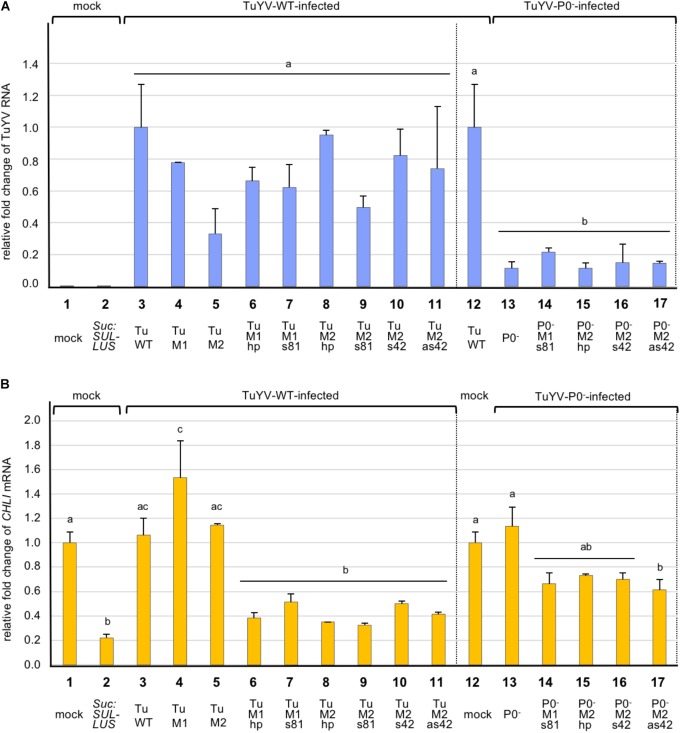
Analysis by real-time RT-PCR of the accumulation of TuYV RNA **(A)**, and *CHLI1* transcripts **(B)**, in plants infected with different TuYV recombinant viruses in *A. thaliana* Col-0 ecotype 19 dpi. The bars represent the fold-change of viral RNA or cellular *CHLI1* transcripts in upper non-inoculated leaves of infected plants, with the TuWT RNA value in infected plants (column 3 and 12, panel A) and *CHLI1* transcript value from mock-inoculated plants (column 1 and 12, panel B) arbitrarily set as references. *Suc:SUL-LUS* corresponds to non-inoculated transgenic plants expressing a hairpin directed against the *CHLI1* gene under the control of the *Suc2* promoter (column 2). TuWT and derived variants (M1, M2, M1hp, M1s81, M2hp, M2s81, M2s42, and M2as42) correspond to plants infected with the relevant viruses (columns 3–11). P0^-^, P0^-^M1s81, P0^-^M2hp, P0^-^M2s42, and P0^-^M2as42 are the mutant viruses in the P0^-^ background (columns 13–17). Data are the mean value ± SE from two to five pools of two plants each (total of 4–10 plants/condition). Statistical analyses were performed using one-way ANOVA following by Tukey’s *post-hoc* test. Different letters above the bars indicate statistically significant differences in the quantification of TuYV RNA **(A)** and *CHLI1* transcripts **(B)** (*p*-value < 0.05), while the same letters indicate no statistically significant differences. The data originate from a single experiment but to make the figure easier to interpret visually, both panels were split with a dotted vertical line and the reference values placed in two locations in each panel, next to the WT-derivative virus values and next to the P0-derivative virus values (panel A, columns 3 and 12; panel B, columns 1 and 12).

Finally we performed real-time RT-PCR to determine the accumulation level of the recombinant viruses in plants. Total RNA was extracted at 19 dpi from non-inoculated leaves of pooled plants and TuYV RNA was quantified using specific primers (Supplementary Table S1). As shown in Figure 4A the multiplication rates of all wild-type-derived viruses were not statistically different from that of TuWT (Figure 4A, bars 3–11). For TuP0^-^ we measured an 8.7 fold reduction compared to TuWT (Figure 4A, compare bars 12 and 13). This confirms previous experiments showing that TuP0^-^ accumulation was reduced by five to seven fold compared to TuWT when tested by ELISA (Ziegler-Graff et al., 1996). The different P0-deficient recombinant viruses showed a similar statistically significant reduction in their viral titer compared to TuWT (Figure 4A, bars 13–17).

### Inhibition of *CHLI1* Expression in Plants Infected With the TuYV Recombinant Viruses

In order to analyze more in depth the fate of the targeted *CHLI1* mRNA in plants infected with the TuYV recombinant viruses, *CHLI1* transcripts were quantified by real-time RT-PCR using specific primers (Supplementary Table S1). *CHLI1* mRNA accumulation was also measured in transgenic *Suc:SUL-LUS* plants and in Col-0 mock-treated plants revealing a reduction of 77.9% of *CHLI1* transcripts accumulation in *Suc:SUL-LUS* plants compared to Col-0 untreated plants (Figure 4B, compare bars 1 and 2). When *CHLI1* expression was analyzed in plants infected with the non-recombinant viruses, no significant effect of viral infection on the *CHLI1* transcript accumulation was noticed for TuWT, TuM2, and TuP0^-^ when compared to the mock-treated plants (Figure 4B, compare bar 1 or 12 with bars 3, 5, and 13). TuM1 infection induced a significant increase of the *CHLI1* mRNA that could not be explained. Conversely, infection with the WT-derived recombinant viruses bearing *CHLI1* inserts resulted in a significant reduction of the *CHLI1* transcript accumulation ranging between 48.8 and 67.5% compared to mock-inoculated plants. Surprisingly although the yellowing phenotype was particularly mild in plants infected with TuM2s42, the effect on *CHLI1* mRNA decrease was similar to that observed with the other TuYV-derived vectors that induced stronger symptoms on infected plants (Figure 4B, compare bar 10 with bars 6–9 and 11 and Figure 3I with Figures 3E–H,J).

Interestingly when compared to the TuWT-based mutants which induced a mean depletion of *CHLI1*-mRNA accumulation of 59.2%, the TuP0^-^-based mutants displayed a more limited reduction of *CHLI1*-mRNA accumulation (32.4%) (Figure 4B, bars 6–11 and 14–17). The decrease in *CHLI1* transcript accumulation was inversely correlated with the virus titer. To summarize, these data show that all recombinant TuWT-*CHLI1* viruses triggered efficient extinction of the *CHLI1* gene, while the recombinant P0-deficient mutants displayed a reduced ability to silence *CHLI1*, likely due to their lower viral titers in the infected plants.

### Analysis of the Genome Stability of the Engineered VIGS Vectors

**FIGURE 5:**
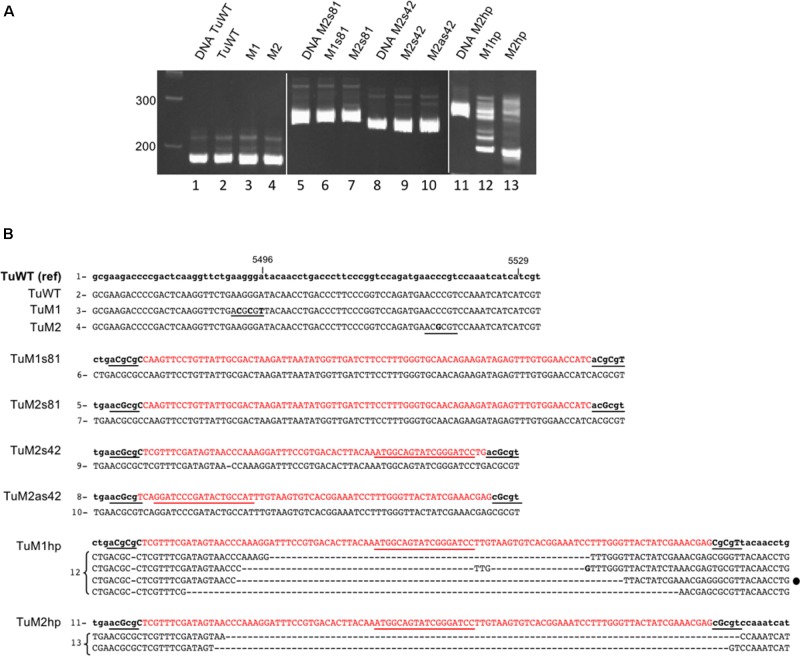
Sequence analysis of recombinant TuYV offspring by RT-PCR. **(A)** Analysis on a 6% polyacrylamide gel of the PCR fragments obtained after reverse transcription performed on RNA extracted 19 dpi from plants agroinfected with TuWT (2) or the different mutants TuM1 (3), TuM2 (4), TuM1s81 (6), TuM2s81 (7), TuM2s42 (9) or TuM2as42 (10), TuM1hp (12), and TuM2hp (13). PCR size controls were produced from the corresponding plasmid DNA (lanes 1, 5, 8, and 11). The first lane at the left represents the size markers in base pairs. **(B)** Sequence analysis of the above described PCR fragments. The reference sequence of each virus (name on the left) is in the upper lane with modified nucleotides in capital letters and the inserted *CHLI1* sequence in red. The numbers at the left of the sequences (1–13) refer to the lanes of the gels shown in panel **A**. The sequence in lower case corresponds to the original viral sequence. The inserted *Mlu*I sites and the *Mlu*I compatible cohesive ends are underlined in black and the nucleotide mutations introduced in the viral genome are in capital letters. The sequences obtained for the offspring of each recombinant virus are shown in capital letters below the reference sequences. For the hp clones, several bands were cut out from the gel and the resulting sequences are indicated. The loop sequence is underlined in red. For TuWT, TuM1 and TuM2 only the progeny sequences were shown as they were identical to their reference sequences. The mismatches with the reference sequence are in bold; (–) means a nucleotide deletion. The black dot at the right side of one TuM1hp sequences represents a particular sequence that was also found in the TuM1hp progeny after aphid transmission (see Supplementary Figure S5).

Viruses with an RNA genome are subject to high mutation rates during replication (Domingo and Holland, 1997; Nagy and Simon, 1997; Duffy et al., 2008). Furthermore, instability is often increased by the introduction of foreign sequences into the viral genome (Nurkiyanova et al., 2000; Zhong et al., 2005; Chung et al., 2007; Majer et al., 2013; Krenz et al., 2016; Ding et al., 2017). Such insertions may reduce virus fitness and induce genome rearrangements and deletions of the foreign sequence after several replication cycles. To investigate potential rearrangements in the genome of the different VIGS-vectors, RNA extracted from systemically infected leaves were analyzed by RT-PCR using oligonucleotides bordering the inserted sequence (Supplementary Table S1). All the amplified PCR fragments obtained from plants infected with the sense or antisense constructs (TuM1s81, TuM2s81, TuM2s42, and TuM2as42) were of expected size (Figure 5A, lanes 6, 7, 9, 10) and showed no or only single nucleotide modifications compared to the original inserted sequences (Figure 5B and data not shown from a second experiment with six other plants infected with TuM2s81). Conversely, analysis of the progeny in plants infected with the hairpin constructs (TuM1hp and TuM2hp) showed several PCR fragments shorter than the reference DNA fragment (Figure 5A, lanes 12 and 13) suggesting a high frequency of deletions within the viral genome. Sequencing of the different fragments revealed deletions ranging from 58 to 89 nts of the 108 nts inserts, confirming the high instability of the hairpin-containing viral genomes (Figure 5B). Surprisingly although the inserted hairpin structure was identical in both recombinant viruses TuM1hp and TuM2hp, the viral progeny analyzed from TuM1hp-infected plants displayed a higher heterogeneity compared to the progeny of TuM2hp-infected plants (Figures 5A,B). Deletions that occurred in the TuM1hp progeny were of different lengths and almost symmetrical relative to the loop. In contrast, deletions in the TuM2hp progeny were larger and covered one entire arm of the hairpin stem and only part of the other side (Figure 5B). Similar results were observed after analyzing additional plants infected with the hairpin recombinant viruses (data not shown). Regarding the TuP0^-^ recombinant constructs, sequence from plants infected with virus containing M2s42, M2as42, or M1s81 inserts displayed an insert stability, in the small sample size tested, similar to what was observed for corresponding TuWT constructs (Supplementary Figure S3).

### Aphid Transmission of the Engineered VIGS Vectors

We next addressed whether the TuYV-derived vectors could be transmitted by aphids and retain their insert. *Arabidopsis* plants agroinfected with either TuM1s81, TuM1hp, or TuWT were used as source plants for virus acquisition by aphids 3 weeks pi. Aphids were then transferred to healthy *Arabidopsis* plants and viral transmission was tested by ELISA 3 weeks later. All plants (10 aphid-inoculated plants/recombinant virus) got infected at a level similar to those of TuWT-inoculated plants (Supplementary Figure S4). All plants aphid-inoculated with TuM1s81 showed a mild but clearly visible vein chlorosis phenotype on developing leaves, suggesting that the viral progeny picked up by aphids in the infected source plants retained the ability to silence the endogenous *CHLI1* gene (Supplementary Figure S5A). Conversely, the viral offspring that was efficiently transmitted by aphids from TuM1hp-infected plants developed symptoms in only 4 out of 10 infected plants and the symptoms remained fainter when compared to those exhibited by the TuM1s81 progeny (Supplementary Figure S5A). This observation suggests that only a fraction of the TuM1hp progeny that was taken up by the aphid from the infected source plants still contained the *CHLI1* insert able to induce silencing.

The genome stability of the recombinant viruses inoculated by the aphids was analyzed by RT-PCR and sequencing as described earlier. Five aphid-inoculated plants were analyzed for each recombinant virus. The viral progeny in all five TuM1s81 infected-plants gave a single PCR fragment of the expected size which sequence was identical to that of the agroinoculated virus (Supplementary Figure S5B) except one nucleotide deletion also found in the source plant that was again observed in the progeny (data not shown). In plants aphid-inoculated with TuM1hp the viral progeny was more heterogeneous and several DNA fragments were obtained after RT-PCR from the offspring RNA (data not shown). Sequencing these PCR fragments revealed large deletions in the *CHLI1* sequence similar to those previously identified in the TuM1hp source plants (Supplementary Figure S5B). In particular one main deletion of 58 nts observed in the M1hp-agroinoculated plants was reproducibly detected in several TuM1hp aphid-infected plants (Supplementary Figure S5B and Figure 5). This experiment confirms the lower stability of the *CHLI1-*hairpin construct when compared to the sense recombinant virus and highlights the remarkable stability of the TuM1s81 mutant even after aphid transmission.

### Silencing of a Phloem Specific Gene *RTM1*

By using TuYV as a VIGS vector to silence the *CHLI1* gene expressed in green tissue^1^ a *CHLI1* transcript reduction ranging between 48.8 and 67.5% was achieved. As TuYV multiplies only in phloem cells we addressed whether silencing of a phloem-specific gene using the TuYV-derived vector would reach a higher extinction level. The *AtRTM1* gene (Restricted-Tobacco etch virus Movement) was chosen since it is expressed only in phloem cells and RTM1 protein accumulates only in companion cells and sieve elements (Chisholm et al., 2001; Cayla et al., 2015). Associated to four other *RTM* genes, *RTM1* provides resistance to long distance movement of several potyviruses in the *Arabidopsis* Col-0 accession (Mahajan et al., 1998; Whitham et al., 1999; Cosson et al., 2012; Revers and Garcia, 2015).

**FIGURE 6:**
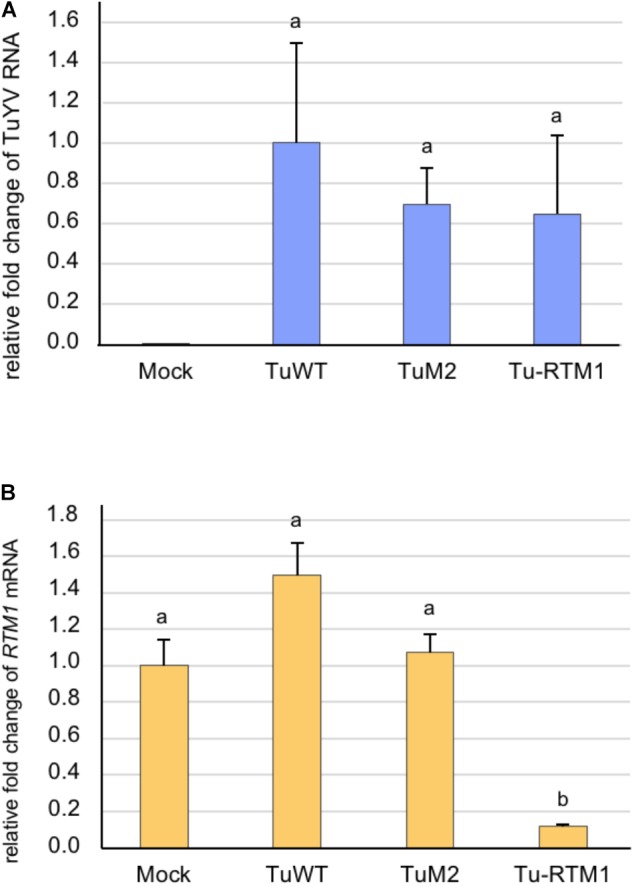
Analysis by real-time RT-PCR of the accumulation of TuYV RNA **(A)** and *RTM1* transcripts **(B)** in infected *A. thaliana* Col-0 plants. Plants were either mock-inoculated (mock) or agroinoculated with TuWT, TuM2, or Tu-*RTM1* and collected 36 dpi. TuM2 is the parental clone in which the *RTM1* sequence was inserted. The bars represent the relative fold-change ± SE of viral RNA or *RTM1* transcripts in upper non-inoculated leaves of individual mock or infected plants. The TuYV value in infected TuWT control plants and the *RTM1* value in the mock-inoculated plants were arbitrarily set to 1 as a reference. Statistical analyses were performed using one-way ANOVA following by Tukey’s *post-hoc* test. Different letters above the bars indicate statistically significant differences for *RTM1* transcripts **(B)** (*p*-value < 0.05) while the same letters indicate no statistically significant differences (**A** and **B**).

An 80 bp fragment from *RTM1* cDNA was inserted in sense orientation into the previously described TuM2 (Figure 1A) resulting in the recombinant Tu-*RTM1*. The control viruses TuWT and TuM2 and Tu-*RTM1* were agroinoculated to Col-0 plants and infection was assessed by DAS-ELISA and real-time RT-PCR on systemically infected leaves of individual plants 4 weeks pi. The recombinant virus was fully infectious as 95% of the inoculated plants were infected (ELISA, data not shown) and the viral titer was in the range of that measured for TuWT and TuM2 (Figure 6A). While accumulation of *RTM1* transcripts in TuWT and TuM2-infected plants was similar to mock-inoculated plants, a statistically significant decrease in *RTM1* mRNA accumulation was observed in Tu-*RTM1*-infected plants (Figure 6B) reaching 88.4% of silencing. No phenotype was observed on Tu-*RTM1*-infected plants (data not shown). This confirms the particularly powerful silencing effect of the TuYV-VIGS vectors when a phloem gene is targeted.

## Discussion

Several studies showed that VIGS technology was effective in plant organs like leaves (Liu et al., 2002), roots (Ryu et al., 2004; Valentine et al., 2004; Bennypaul et al., 2012), tubers (Faivre-Rampant et al., 2004), flowers (Chen et al., 2004; Liu et al., 2004; Constantin et al., 2004) or fruits (Fu et al., 2005; Senthil-Kumar and Mysore, 2011). Phloem-specific silencing has been recently reported using a geminivirus to specifically knockdown the expression of the Phytoene desaturase gene (*PDS*) in the vasculature of *N. benthamiana* (Kushwaha and Chakraborty, 2017). In the present work we have developed a novel tool adapted to poleroviruses to perform VIGS in the vascular tissue. By introducing small host gene sequences into the TuYV genome, phloem specific and robust silencing was achieved for two genes with different expression patterns, *CHLI1* expressed in all cells including phloem cells and *RTM1* which is phloem-restricted.

Until recently poleroviruses were refractory to stable insertions of foreign sequences into their genome. Nurkiyanova et al. (2000) constructed a GFP-tagged Potato leafroll virus that was unstable in systemically infected leaves as confirmed by large deletions that occurred in the viral genome and associated with absence of fluorescence. Boissinot et al. (2017) reported the first engineered polerovirus that was able to move to non-inoculated leaves and was visualized by fluorescence microscopy after inserting the EGFP gene in the TuYV genome. The recombinant clone was, however, less infectious than the wild-type virus, likely due to the partial deletion of ORF5 generated to introduce the exogenous sequence. In addition, the viral progeny was prone to genomic rearrangements leading to the loss of the EGFP sequence integrity. These modifications occurred in a host-specific manner and were particularly frequent in *A. thaliana*. Based on these findings and in order to produce a silencing vector that would remain stable over time, the maximum insert size introduced into the wild-type TuYV genome was fixed to 108 nts. All the recombinant TuYV VIGS vectors obtained were infectious in *A. thaliana*. Addition of a foreign sequence into the 3′ untranslated region of the TuYV genome at any of the two inserted restriction sites did not significantly alter viral accumulation in systemically infected leaves. Importantly all TuYV *CHLI1*-recombinant viruses, whatever the type of insert, were able to induce silencing of the cognate gene in infected plants.

A 42 nts fragment bearing 100% identity with the target *CHLI1* gene and inserted into the viral genome in a sense or antisense orientation was found sufficient to induce silencing of the gene without altering the genome stability in the limited number of s42 and as42 TuWT and TuP0^-^ constructs analyzed (Figure 5 and Supplementary Figure S3). A stronger visual silencing effect was obtained either by inserting the same sequence as an IR with a small loop (hp) or by introducing a longer and different sequence of *CHLI1* (81 nts) in a sense orientation. The s81 construct, in the TuM1 or TuM2 background, was remarkably stable in sequence and length over a longer period compared to the hp construct, and moreover, at least in the TuM1 background, remained stable after aphid transmission. These results suggest that the TuYV genome instability observed with the hairpin insert is directly related to the highly structured RNA loop which likely causes pausing of the viral RNA-dependent RNA polymerase during replication (Nagy and Simon, 1997), and not to the inserted sequence *per se*.

Similar VIGS experiments performed with short inserts introduced in different VIGS vectors gave variable results when silencing robustness was considered. Using various TYMV-*PDS* (Turnip yellow mosaic virus-*PDS*) constructs, Pflieger et al. (2008) showed that a 2 × 37 nts long IR sequence was able to efficiently trigger the photobleaching phenotype while a 45 nts long sense or antisense sequence could not. The phenotype could be observed up to 6–8 weeks after inoculation suggesting that the recombinant viral genome was stable during this period. Such differences in exogenous hairpin stability in TuYV- or TYMV-derived vectors could be related to the insertion site into the viral genome that may be more or less tolerant to highly structured foreign sequences. A study performed with several recombinant Tobacco mosaic virus and Barley stripe mosaic virus (BSMV) constructs reported stronger silencing phenotypes using short hairpins (40 or 60 nts long) rather than similar sense and antisense constructs sequences (Lacomme et al., 2003) but the issue of genome stability over time was not addressed. VIGS assays with BSMV containing PDS fragments of increasing lengths (128–584 nts) showed that the stability of the photobleaching phenotype was dependent on the insert length, deletions occurring more frequently with longer inserts (Bruun-Rasmussen et al., 2007). Sense and antisense versions gave similar levels of silencing. Conversely using a double stranded DNA pararetrovirus (Rice tungro bacilliform virus) Kant and Dasgupta (2017) showed that derived viral constructs containing antisense or hairpin insertions induced higher silencing compared to sense oriented constructs. It seems therefore that each virus reacts with its own specificities regarding the type of insert used to generate a VIGS effect.

When considering the gene silencing efficiency of the TuYV-derived vectors, we observed that the Tu*-CHLI1* vectors induced slightly lower reduction rates of *CHLI1* transcripts accumulation (49–67%) compared to the silencing level measured in stable transformed transgenic *Suc:SUL-LUS* plants (78%). In these latter plants *CHLI1* silencing is driven by a *SULFUR* hairpin under the control of a companion cell specific promoter (Himber et al., 2003). This proves that gene silencing efficiency provided by the VIGS methodology is comparable to that observed in transgenic plants. Moreover VIGS can be set up in a shorter time frame and without the need of genetically transformed plants. Surprisingly although visual differences were observed among the vein yellowing phenotypes in plants infected with the different recombinant viruses, no significant variations in the *CHLI1-*mRNA accumulation were observed. This attests that visual photobleaching is not always perfectly correlated with the level of transcript accumulation as previously observed in the case of silencing experiments targeting the *PDS* gene (Ratcliff et al., 2001; Lacomme et al., 2003; Bruun-Rasmussen et al., 2007). This effect may be related to the half-life of the proteins targeted by silencing.

It is noteworthy that the silencing efficiency for the phloem-specific *RTM1* gene using Tu-*RTM1* reached nearly 90%, highlighting the robustness and the usefulness of TuYV vectors. For a non-phloem specific gene (*CHLI1*) the knockdown level obtained with the phloem-specific TuYV vector is in the same range (49–67%) as those obtained with several non-phloem specific VIGS systems (70% for a Potato virus X vector (Ratcliff et al., 2001), 61–87% for TMV-based vectors (Lacomme et al., 2003), 75–90% for TYMV-PDS (Pflieger et al., 2008), 61% for a geminivirus Tomato mottle virus-*chI*I (Esparza-Araiza et al., 2015) and 40–80% for a pararetrovirus Rice tungro baciliform virus-*chl*H (Kant and Dasgupta, 2017). The paradoxical important reduction of non-phloem *CHLI1* transcripts in plants infected with phloem-limited TuYV recombinants can be explained by the spread of the vsiRNAs, produced in infected phloem cells, into the surrounding cells, allowing the silencing process to extend beyond the sites of initiation, if not countered by the RSS P0 (see below).

Until the present study, the mechanism of action of the RSS P0 was essentially studied in ectopical conditions and not in the context of a viral infection. Former studies performed with P0 in the absence of virus infection provided evidence that P0 functions downstream of siRNA production by Dicer enzymes (Baumberger et al., 2007; Bortolamiol et al., 2007). P0 was shown to direct the degradation of AGO1 protein by autophagy (Derrien et al., 2012) and AGO1 targeting by P0 occurs only before the assembly into RISC (Csorba et al., 2010). As P0 does not possess RNA binding activity (Zhang et al., 2006; Csorba et al., 2010) the vsiRNA produced in infected cells are likely to be able to traffic to non-infected neighboring cells and become loaded into the adjacently located RISC. The detected vsiRNA co-immunoprecipitated with AGO1 proteins may be those that moved out of the infected phloem cells where TuYV is confined, into non-phloem cells or non-infected phloem cells. This supports the idea that the vein yellowing phenotype observed on plants infected by the recombinant TuWT-*CHLI1* vectors reflects the silencing signal produced in the infected cells which spread to a limited number of surrounding non-infected cells (Himber et al., 2003; Melnyk et al., 2011). Our observations that P0-deficient recombinant viruses exhibited milder and not enhanced vein clearing phenotypes in infected plants compared to the corresponding wild-type VIGS viruses, confirm that P0 does not interfere with the production nor the cell-to-cell movement of the silencing signal and that P0 does not impede performances of the TuYV silencing effect.

Monitoring the spread of *Luteoviridae* infection *in planta* has always been a challenge for the aforementioned reasons. With the recent development of a systemically infecting and fluorescent TuYV clone, Boissinot et al. (2017) overcame the first step in obtaining a viral vector that can be traced *in vivo*. The fluorescence-based technology requires, however, specific facilities and equipment that are not available in all laboratories particularly when large scale experiments are considered. The recombinant TuYV-*CHLI1* clones described here represent therefore the first examples of undeleted infectious viral clones of members of the *Luteoviridae* family for which infection progression *in planta* can be rapidly and easily monitored, both spatially and temporarily, by visual observation of a vein clearing phenotype. Importantly TuYV, like other poleroviruses, does not provoke any noticeable disease symptoms in *A. thaliana*, an essential feature for large scale phenotype screening. This powerful reporter system reported here for TuYV can be easily adapted to other polerovirus infectious clones and to any other host, provided that the target sequence is customized for the relevant host. By substantially enhancing discrimination of infected plants from non-infected plants with a visible marker, this technology will pave the way to large scale screenings for polero- or luteovirus resistance genes. For instance by using a VIGS vector derived from the geminivirus Cabbage leaf curl virus carrying a *CHLI1* insert to screen Arabidopsis accessions, Reyes et al. (2017) recently identified a line immune to two different genera of *Geminiviridae*. This technology can considerably speed up the process required to assess plant resistance, as reported in a screen of cassava cultivars. Beyene et al. (2017) modified a geminivirus (East African cassava mosaic virus) by inserting a fragment of a cassava SPY gene in its genome which caused severe shoot tip necrosis followed by the death of susceptible plants only. Finally, as the TuYV VIGS vector carrying the sense-oriented insert retained its ability to develop the vein clearing phenotype after aphid transmission, aphids could be used as an efficient means to inoculate the recombinant viruses in natural conditions to easily screen for plants resistant to aphids. Absence of VIGS symptoms would thus indicate either the inability of the aphid to inoculate the virus or the resistance to the virus. Such an aphid-transmissible VIGS vector could be developed for screenings in plant species that are recalcitrant to agroinoculation.

Several studies developed large scale forward genetic screens using VIGS to suppress specific transcripts and identify genes involved in plant defense response to various pathogens (Peart et al., 2005; Cai et al., 2006; Mantelin et al., 2011; Nagaraj et al., 2016; Zhang et al., 2016). The TuYV VIGS vectors could provide a means to specifically silence phloem genes and decipher the virulence strategies of phloem-limited pathogens (Bendix and Lewis, 2016). In summary polerovirus-based VIGS vectors may have an additional value in functional genomic studies, thus providing novel perspectives in high throughput plant phenotyping screenings for various traits including virus and aphid resistance and also for functional analyses of significant phloem genes.

## Author Contributions

DB-B, VZ-G conceived and designed the experiments. DB-B, BM, SC, KH, DS, AA, VB, and VZ-G performed all the experiments. DB-B, BM, AA, FR, VB, and VZ-G analyzed the data. FB, FR performed the statistical analysis. DB-B, VZ-G wrote the paper. All authors contributed to manuscript revision, read and approved the submitted version.

## Conflict of Interest Statement

The authors declare that the research was conducted in the absence of any commercial or financial relationships that could be construed as a potential conflict of interest.

## Footnotes

1 http://bar.utoronto.ca/efp2/Arabidopsis/Arabidopsis_eFPBrowser2.html
