# Supplementary material for: Phloem-Triggered Virus-Induced Gene Silencing Using a Recombinant Polerovirus
Source: Front Microbiol. 2018 Oct 23;9:2449. doi: 10.3389/fmicb.2018.02449 (PMC6206295; doi:10.3389/fmicb.2018.02449)
Supplement: Supplementary file 1 [file Table_1.docx]

**Table S1.** List of oligonucleotides used.

Mutagenic nucleotides to generate M2 site are underlined.

| primer name | primer sequence |
| --- | --- |
| Mlu2-Dir | GATGAACGCGTCCAAATCATCATCG |
| Mlu2-Rev | GGACGCGTTCATCTGGACCGG |
| Tu-5351-Fw | ATCTCGAGCTCCGCCAAGTCGGAG |
| Bin-Tu-Rev | ATAGATCTCTATGACCATGATTACGCC |
| CHLI1-508-Fw | ATGCGCGCCAAGTTCCTGTTATTGCG |
| CHLI1-588-Rev | TAACGCGTGATGGTTCCACAAACTC |
| CHLI1-42-1 | CGCGCTCGTTTCGATAGTAACCCAAAGGATTTCCGTGACACTTACAAATGGCAGTATCGGGATCCTGA |
| CHLI1-42-2 | CGCGTCAGGATCCCGATACTGCCATTTGTAAGTGTCACGGAAATCCTTTGGGTTACTATCGAAACGAG |
| CHLI1-hp1 | GATCCTTGTAAGTGTCACGGAAATCCTTTGGGTTACTATCGAAACGAG |
| CHLI1-hp2 | CGCGCTCGTTTCGATAGTAACCCAAAGGATTTCCGTGACACTTACAAG |
| RTM1-276-Fw | ACTACGCGTCAATCCTCACATGAGGTCTC |
| RTM1-357-Rev | TCAACGCGTCGTTGCTCGAACCGGAGG |
| Tu-5453-Fw | AGAATCACTTCTAGCTGGCGA |
| Tu-5641-Rev | ACACCGAAGTGCCGTAGG |
| Tu-3694-Fw | AAGACAATCTCGCGGGAAG |
| Tu-3830-Rev | GGAGACGAACTCCAAAATGAC |
| Tu-4942-Rev | GTGTCAGAACCCCCACTAGC |
| Tu-5280-Fw | GCTGTGTTAGACGGTCGCAAG |
| CHLI1-245-Fw | TCCAAATAAGGCCAAAGAAGAACAG |
| CHLI1-340-Rev | ACTTCCCTACTACTTGTTCAGTAGA |
| RTM1-189-Fw | CGGCAACATGTTTGACGTTAT |
| RTM1-319-Rev | CGCTAGTGTTGGTATTGAATTTGA |
| SAND-Fw | AACTCTATGCAGCATTTGATCCACT |
| SAND-Rev | TGATTGCATATCTTTATCGCCATC |
| EF1α-Fw | CACCACTGGAGGTTTTGAGG |
| EF1α-Rev | TGGAGTATTTGGGGGTGGT |
