# Supplementary figures and images for: Phloem-Triggered Virus-Induced Gene Silencing Using a Recombinant Polerovirus
Source: Front Microbiol. 2018 Oct 23;9:2449. doi: 10.3389/fmicb.2018.02449 (PMC6206295; doi:10.3389/fmicb.2018.02449)

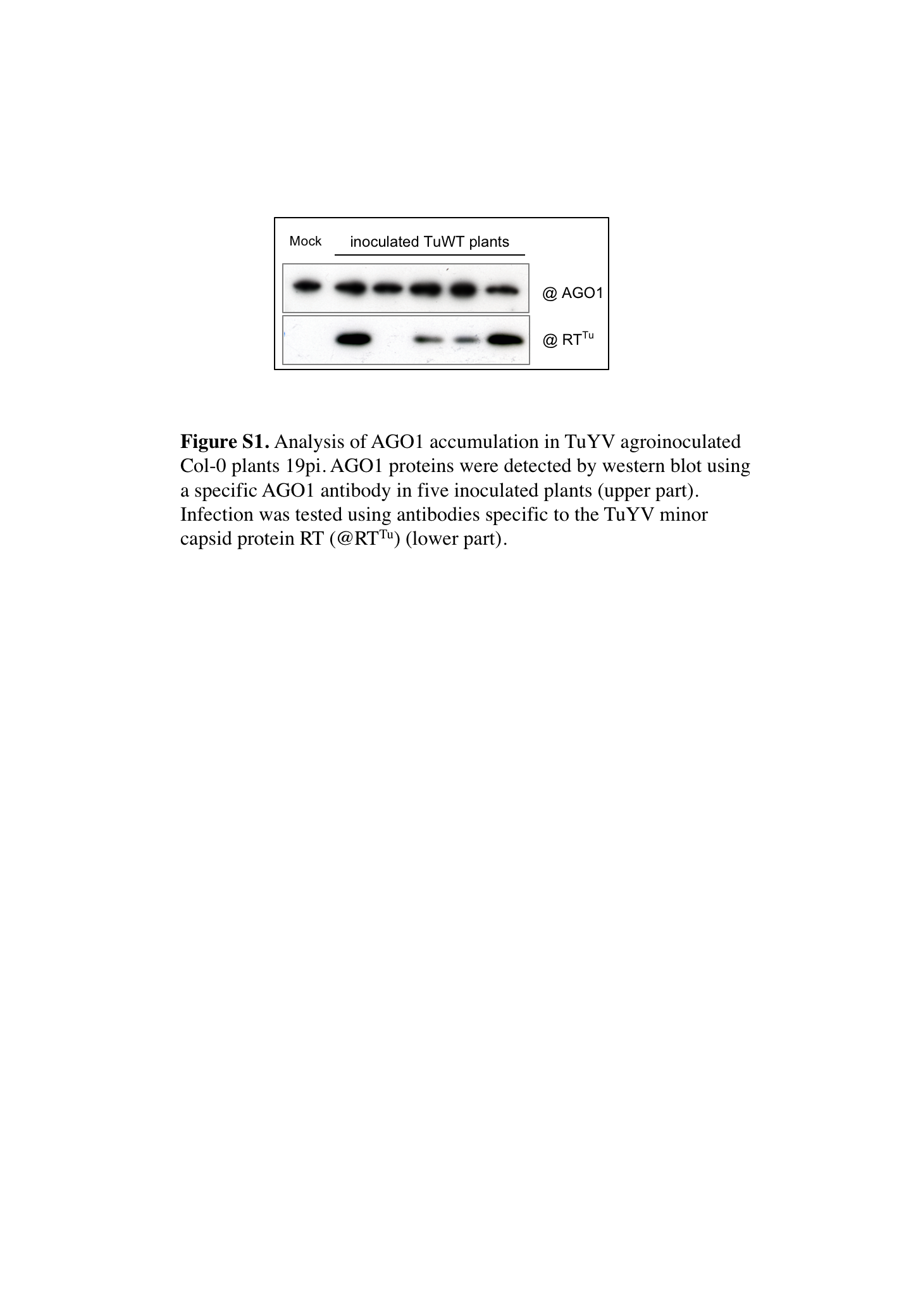

Supplement: Supplementary file 2 [file Image_1.TIFF]

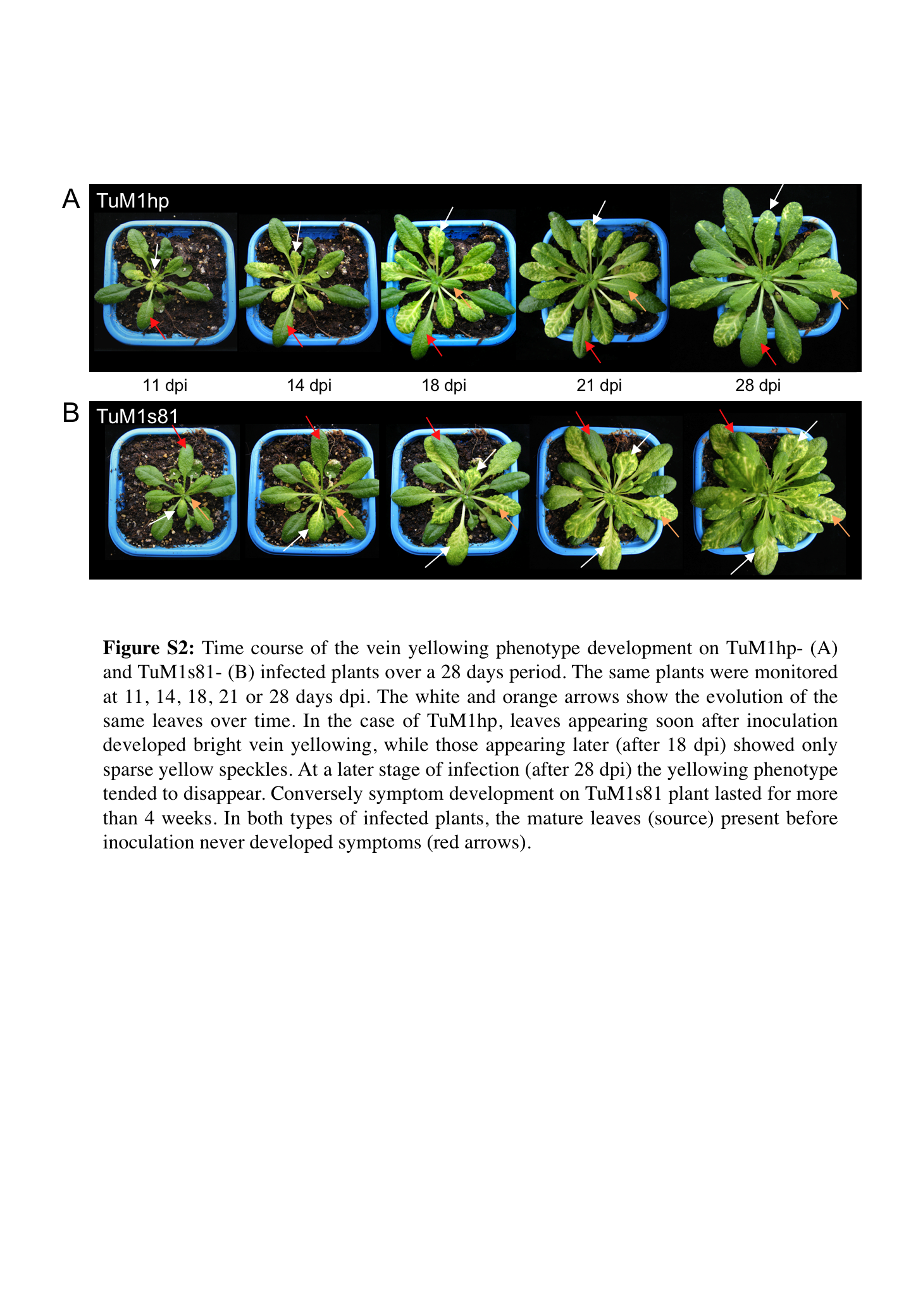

Supplement: Supplementary file 3 [file Image_2.TIFF]

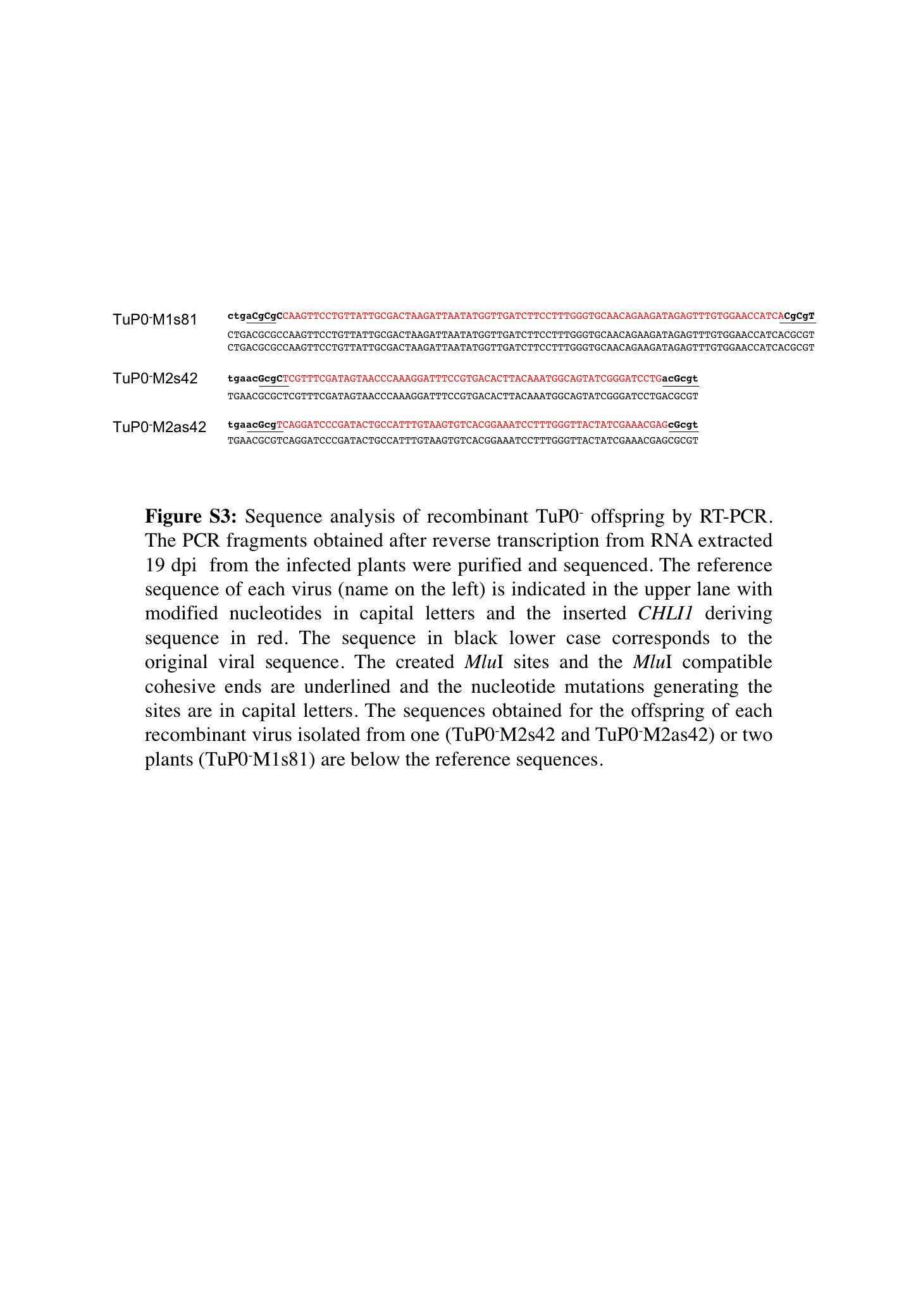

Supplement: Supplementary file 4 [file Image_3.TIFF]

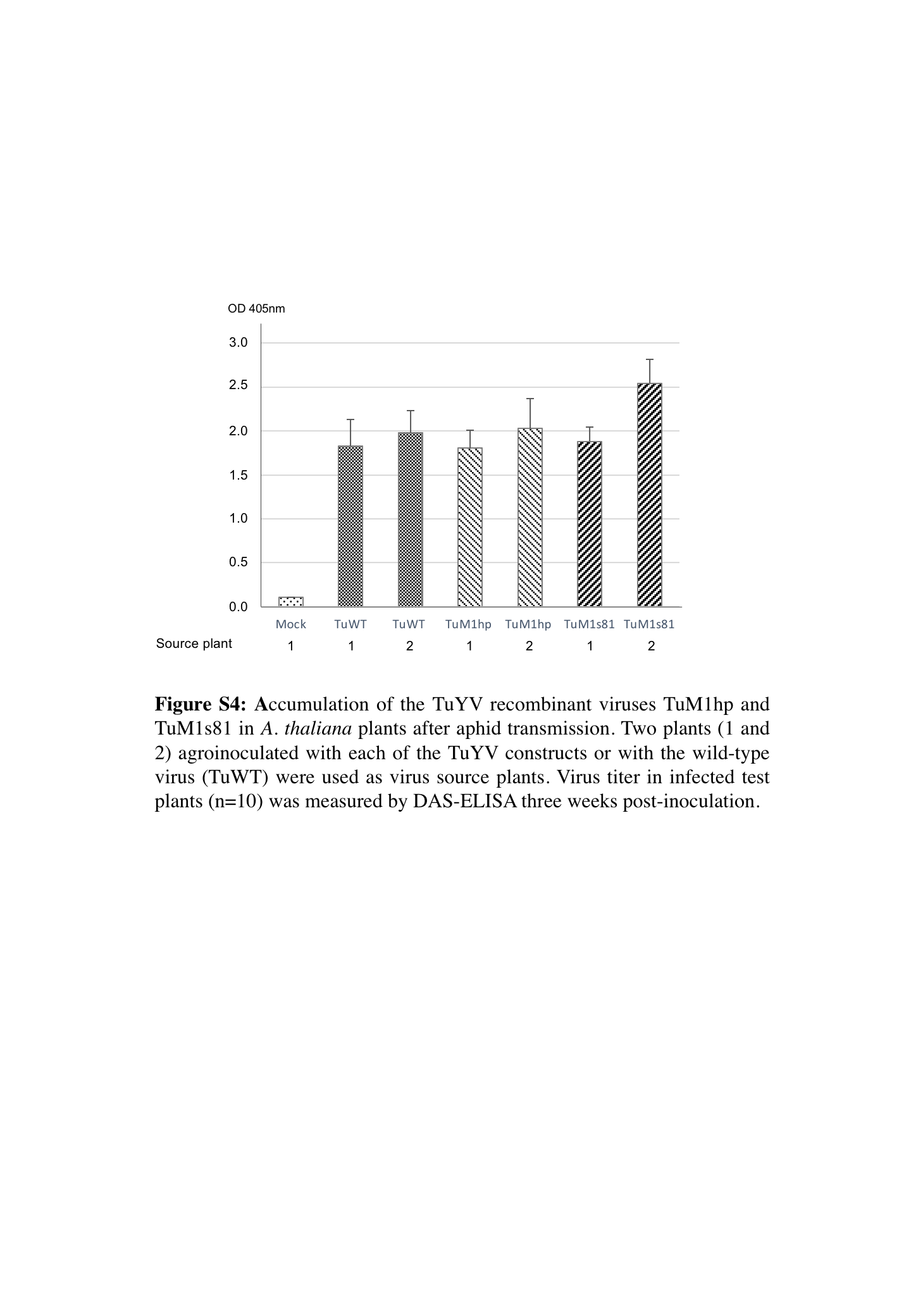

Supplement: Supplementary file 5 [file Image_4.TIFF]

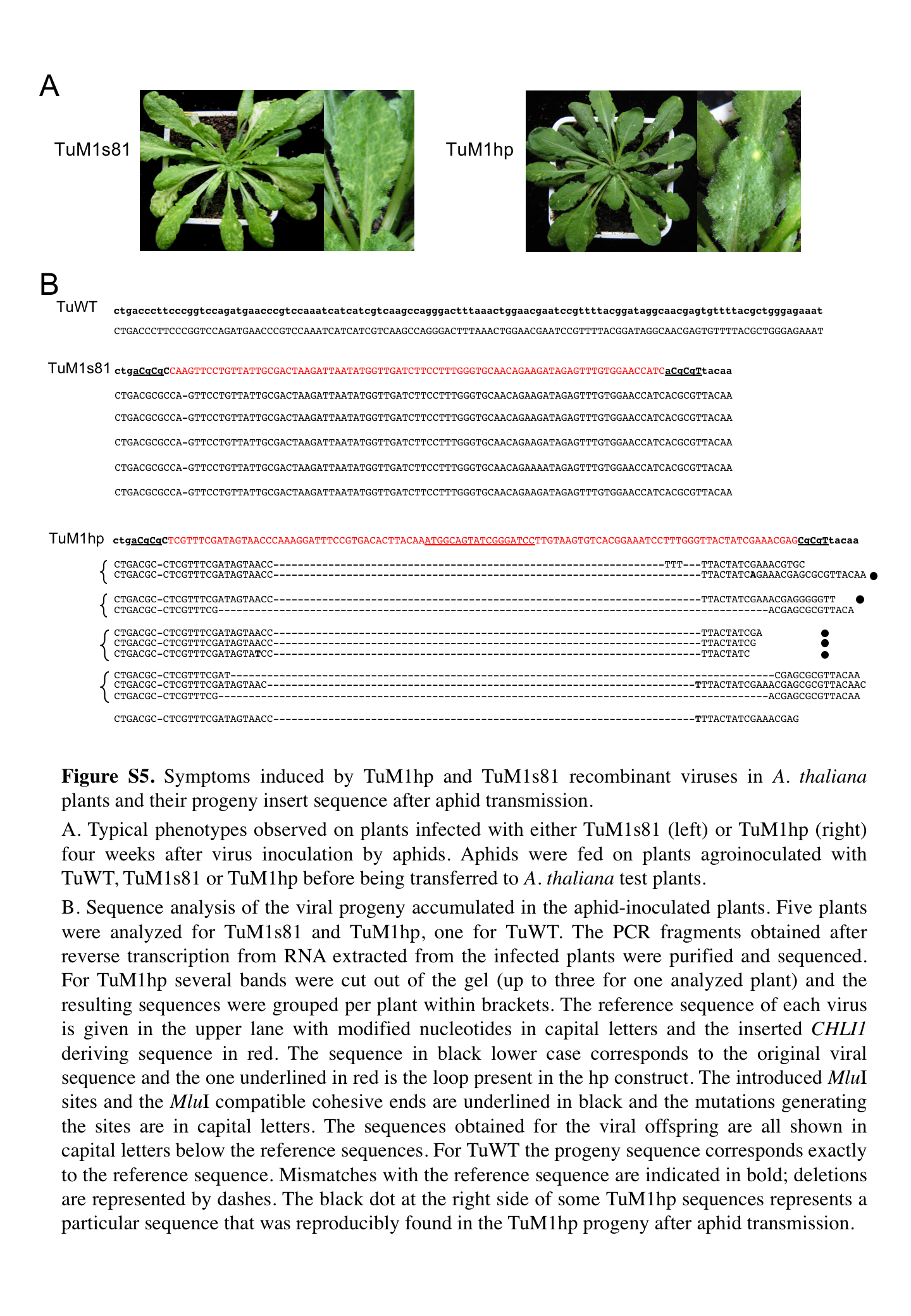

Supplement: Supplementary file 6 [file Image_5.TIFF]
